# Epigenetic remodelling of brain, body and behaviour during phase change in locusts

**DOI:** 10.1186/2042-1001-1-11

**Published:** 2011-07-26

**Authors:** Malcolm Burrows, Stephen M Rogers, Swidbert R Ott

**Affiliations:** 1 Department of Zoology, University of Cambridge, Downing Street, Cambridge CB2 3EJ, UK

## Abstract

The environment has a central role in shaping developmental trajectories and determining the phenotype so that animals are adapted to the specific conditions they encounter. Epigenetic mechanisms can have many effects, with changes in the nervous and musculoskeletal systems occurring at different rates. How is the function of an animal maintained whilst these transitions happen? Phenotypic plasticity can change the ways in which animals respond to the environment and even how they sense it, particularly in the context of social interactions between members of their own species. In the present article, we review the mechanisms and consequences of phenotypic plasticity by drawing upon the desert locust as an unparalleled model system. Locusts change reversibly between solitarious and gregarious phases that differ dramatically in appearance, general physiology, brain function and structure, and behaviour. Solitarious locusts actively avoid contact with other locusts, but gregarious locusts may live in vast, migrating swarms dominated by competition for scarce resources and interactions with other locusts. Different phase traits change at different rates: some behaviours take just a few hours, colouration takes a lifetime and the muscles and skeleton take several generations. The behavioural demands of group living are reflected in gregarious locusts having substantially larger brains with increased space devoted to higher processing. Phase differences are also apparent in the functioning of identified neurons and circuits. The whole transformation process of phase change pivots on the initial and rapid behavioural decision of whether or not to join with other locusts. The resulting positive feedback loops from the presence or absence of other locusts drives the process to completion. Phase change is accompanied by dramatic changes in neurochemistry, but only serotonin shows a substantial increase during the critical one- to four-hour window during which gregarious behaviour is established. Blocking the action of serotonin or its synthesis prevents the establishment of gregarious behaviour. Applying serotonin or its agonists promotes the acquisition of gregarious behaviour even in a locust that has never encountered another locust. The analysis of phase change in locusts provides insights into a feedback circuit between the environment and epigenetic mechanisms and more generally into the neurobiology of social interaction.

## Background

One of the central questions in biology is how disparate developmental processes are dynamically regulated by the interplay between genomic information and environmental drivers to produce coherent phenotypes. The environment may exert a profound effect on development by engaging mechanisms that tailor gene expression in the light of experience to produce phenotypes that are better able to deal with the specific conditions encountered. This extends the ecological reach of individual organisms without altering their underlying genome (epigenetic remodelling). Such phenotypic plasticity is common in nature and may affect physiology [1,2], morphology [3] and behaviour [4,5]. The extent and rate at which an animal may undergo epigenetic remodelling is highly variable and depends on the species, the developmental stage and the body system affected. In extreme cases, the genome in effect contains the potential to develop several different kinds of animal. On the other hand, more restricted phenotypic plasticity in the nervous system is a nearly universal occurrence, in the guise of learning and memory. Some animals may undergo neuronal plasticity at any stage in their lifetimes, although in others, particular kinds of learning are restricted to certain life stages. A range of explanations for phenotypic plasticity have been offered at the mechanistic [6,7], population genetic, and evolutionary levels [8,9], but a synthesis awaits.

Behaviour is a particularly labile feature of the phenotype, but the plasticity responsible for altering it extends beyond the nervous system. Behavioural modification can involve alterations not only in neurons but also in sensory structures, muscles and the skeleton, all of which may be limited in the rate or extent they can change. For example, neuronal plasticity can proceed more quickly than muscle growth or skeletal remodelling. How, then, does the nervous system compensate for this lag? Can it produce a motor output in the short term that makes the best use of the available motor system, whilst the muscles and skeleton adapt more slowly to the new behavioural requirement? At the cellular level, the challenge is to identify mechanisms that translate experience into both short-term and long-lasting phenotypic modifications coordinated across whole suites of behaviour and integrated with altered motor systems and morphology to produce coherent behavioural syndromes. All the while the animal must remain fully functional as it undergoes these changes.

One of the most striking and changeable facets of the environment is the presence and actions of other members of the same species, which can be potent drivers of behavioural and other phenotypic change, whether as potential mates or competitors, or indirectly through the effects they have on shared habitats. These socially induced forms of phenotypic plasticity are often triggered by specific stimuli and may operate through distinct mechanistic pathways, such as the action of sex hormones or biogenic amines regulating agonistic and other social interactions [10-12].

**Figure 1:**
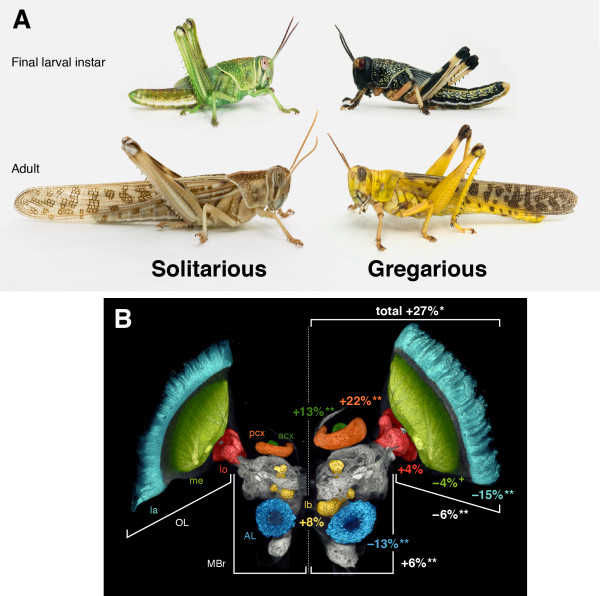
**Body and brain changes between solitarious and gregarious locusts**. **(A) **Differences in colouration and body size and shape in final larval instar and adult solitarious and gregarious locusts are shown. **(B) **Differences in brain size and proportions of particular regions in solitarious (left) and gregarious (right) brains of adult locusts are shown. Data from [13]. Regions in the midbrain (MBr) include the olfactory antennal lobe (AL) and three neuropils in the mushroom body: the olfactory primary calyx (pcx), the gustatory accessory calyx (acx) and the multimodal lobes (lb). The optic lobe (OL) comprises three successive visual neuropils: the lamina (la), the medulla (me) and the lobula (lo). Absolute total brain size is 27% larger in gregarious locusts. The remaining numbers refer to the differences in proportions of different brain regions relative to total brain size. Positive numbers indicate that a region is disproportionally larger in gregarious locusts than in solitarious locusts (**P < 0.01; *P < 0.05; +*P *< 0.1).

Our preferred model for analysing both the mechanisms and functional consequences of phenotypic plasticity is the desert locust (*Schistocerca gregaria*). These insects show one of the most dramatic examples of phenotypic plasticity of any animal, which encompasses both a socially driven mechanism and multifaceted changes in behaviour and motor systems. They can change reversibly between two forms that differ so extensively in appearance (Figure 1A), behaviour, physiology, neurochemistry and morphology [13] that they were once thought to be different species. This transformation, or *phase change*, is driven by fluctuations in population density [14,15]. At low densities, locusts occur in the *solitarious phase*. The whole biology of solitarious locusts is governed by the need to be inconspicuous, and they are cryptic in colouration and behaviour. They walk with a slow, creeping gait, fly predominately at night, have a restricted diet and actively avoid other locusts, thus maintaining their low population density. They have long wings and jumping hind legs, large eyes and long antennae.

When sheer population density and increasingly patchy food resources force solitarious locusts together despite their natural aversion to each other, a far-reaching transformation is triggered that results in the *gregarious phase*. The biology of gregarious phase locusts is dominated by the needs of group living. There is intense competition for resources and a considerably greater sensory complexity in the environment brought about by living in the midst of a constantly moving throng of other animals. Gregarious locusts walk with a rapid, upright gait, fly during the day, have a broad diet and, most importantly, are attracted to other locusts. With inconspicuousness no longer an option, they have bright warning colours, which advertise that they eat small quantities of poisonous plants to make themselves distasteful to predators. They have shorter wings and hind legs, smaller eyes and antennae, but more close-proximity touch and taste receptors. Gregarious locusts can aggregate into vast migratory swarms through their mutual attraction, which still pose an enormous economic and social threat to many parts of the world.

This transformation process can be induced in the laboratory, and, with suitable husbandry techniques [16], locusts of either phase can be readily obtained. In the past few years, considerable progress has been made in unravelling the time course of phenotypic change and the stimuli that induce it, and we are beginning to understand the molecular mechanisms driving the early stages of this transformation. The sheer extent of behavioural change provides a rich substrate with which to perform comparative analyses of how neuronal circuits organise behaviour. Such research is considerably facilitated by the detailed information already available about the structure and function of the nervous systems of locusts, which ranges from its gross anatomy [17] to the cellular physiology [18] of individual neurons and circuits. In the thoracic ganglia, we know much about the connections and actions of sensory neurons on different populations of spiking and nonspiking interneurons and, in turn, their effects on small groups of individually identifiable motor neurons. We also know of several different neuromodulatory neurons that can modify the flow of information through these neuronal networks [18]. Locusts therefore present an unparalleled model system in which to analyse the mechanisms, constraints and consequences of epigenetic modification of the phenotype in terms of the actions of individual neurons and known circuits.

### Differences in overall brain structure

Given the scale of the change in their lifestyle and behaviour, how is this manifested in the nervous system of solitarious and gregarious locusts? The extent of change can be gauged by comparing the relative and absolute sizes of different brain regions between the two phases [13] (Figure 1B). The most obvious difference is that the brains of long-term gregarious locusts are on average 30% larger than those of long-term solitarious locusts, even though the latter have larger bodies. Moreover, different regions occupy very different proportions of the brain in the two phases. Despite having smaller brains, solitarious locusts have disproportionally large primary visual and olfactory neuropils, indicating that they invest more in lower-level sensory processing. The solitarious lifestyle entails greater activity at lower light levels and the need to detect stimuli over greater distances, given the increased individual predation risk, both of which would favour sensitivity in sensory systems. By contrast, gregarious locusts have a larger midbrain to optic lobe ratio, and within both the visual and olfactory systems, higher integration centres are disproportionately larger than the primary sensory neuropils. This reaches an extreme in the olfactory system, where the ratio of the primary calyx of the mushroom body (a second-order olfactory neuropil) to the antennal lobes (the primary olfactory neuropil) is 50% larger in gregarious locusts. Other regions of the mushroom body are also larger in gregarious locusts, but scale in proportion to their generally larger brains. This echoes differences in mushroom body size in different castes and/or social roles in bees, wasps and ants [19-21]. The central complex, an important multimodal sensory and sensory-motor integration centre, is also considerably larger in gregarious locusts. This suggests that the larger brains of gregarious locusts prioritize higher levels of integration to support living in dense and highly mobile swarms dominated by intense competition from other locusts and the behavioural demands of being a generalist forager.

### Changes in individual neurons and connections

To illustrate the differences that occur in individual neurons in the two phases, we draw on two examples of identified interneurons that carry information from receptors on the head to the thoracic ganglia, where motor patterns are organised.

**Figure 2:**
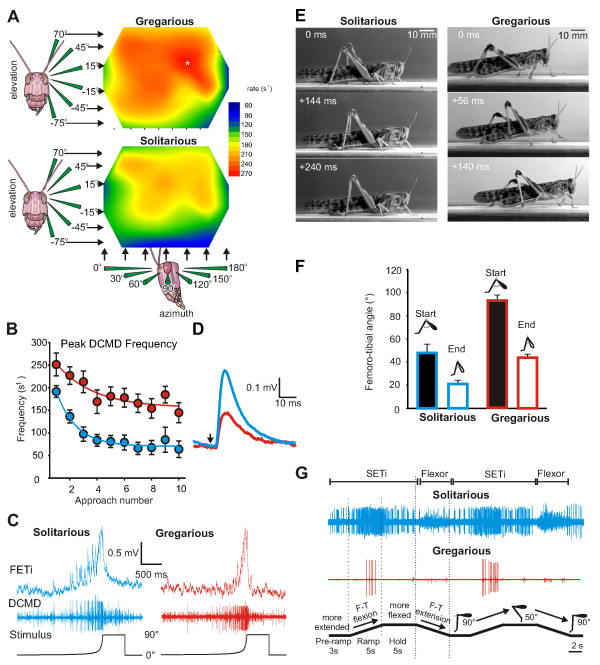
**Differences in identified neurons and neuronal circuits between solitarious and gregarious locusts**. **(A) **The receptive field organization of the visual looming detector neuron DCMD across a visual hemisphere in solitary and gregarious locusts is shown. Maximum spike rates of DCMD are colour-coded. Data from [23]. **(B) **The DCMD of solitarious locusts (blue) shows more pronounced habituation to a visual stimulus repeated at 45-second intervals than that in gregarious locusts (red). **(C) **Compound EPSP response in FETi (upper trace) evoked by activity in the DCMD (middle trace) following exposure to a looming stimulus (angular subtense to 90°, lower trace) is shown. **(D) **EPSPs evoked by individual DCMD spikes in FETi are twice the amplitude in solitarious locusts (blue) compared to those in gregarious locusts (red). Data for **(B) **through **(D) **are from [24]. **(E) **Individual frames from walking sequences of solitarious and gregarious locusts are shown. **(F) **Histograms of excursions of the hind femorotibial joint during a walking step by solitarious and gregarious locusts are shown. **(G) **SETi shows tonic activity and a consistently greater response during an imposed resistance reflex of the hind femorotibial joint in solitarious locusts compared to gregarious locusts. Data for **(E) **through **(G) **are from [28].

The descending contralateral movement detector (DCMD) is a large intersegmental interneuron that responds to looming visual stimuli and signals that an object is on course to collide with the locust [22]. The receptive field organization of this neuron is similar in both phases, spanning the entire visual hemisphere [23], but with a large central region of nearly equal response spanning 120° × 60° (Figure 2A). Within this region, the DCMD of gregarious locusts generates more spikes at a higher frequency than that of solitarious locusts to the same visual stimulus [24]. Moreover, there is a small caudolateral focus of even greater responsiveness found only in gregarious locusts. In the periphery of the receptive field, the response to looming stimuli is weaker and more similar in both phases.

The DCMD of gregarious locusts shows markedly less habituation to repeated presentations of the same stimulus in the central region of the eye, showing only a 34% reduction in spike rate to the seventh presentation compared to a 66% reduction in solitarious locusts [25] (Figure 2B). This should help gregarious locusts to maintain the responsiveness required to avoid colliding with other locusts in a swarm. By contrast, the escape behaviour of the more vulnerable solitarious locusts should be predicated on their need to escape once and then hide again, relying on their camouflage.

The DCMD makes a monosynaptic connection onto an identified leg motor neuron, the fast extensor tibiae (FETi). Despite looming objects eliciting stronger spike responses in the DCMD of gregarious locusts, the resultant synaptic drive to the FETi has the same maximum amplitude in both phases (Figure 2C). In part, this is because individual excitatory postsynaptic potentials (EPSPs) recorded in the FETi in gregarious locusts are only half the amplitude of those in solitarious locusts [24] (Figure 2D). Repeated DCMD spikes in gregarious locusts result in much stronger facilitation of the EPSPs in the FETi. This amplifies the strength of response to genuine looming stimuli (which elicit a strong response in the DCMD) over that to nonlooming visual stimuli. The combination of a homeostatic modulation of the response and a nonlinear synaptic transformation of timing tunes the DCMD-FETi synapse so that gregarious locusts respond earlier than solitarious locusts to small moving objects. Neighbouring locusts in a swarm are the most likely objects to be avoided.

The tritocerebral commissure giants (TCGs) are a pair of identified neurons that convey signals from wind-sensitive hairs on the head to flight motor neurons in the thorax [26]. Gregarious locusts have a lower threshold for initiating flight to wind stimuli compared with solitarious locusts, but have fewer wind-sensitive hairs on the head [27]. The spike frequency in these interneurons in response to wind stimuli is correspondingly higher in gregarious than in solitarious locusts, but not when flight is actually initiated. This suggests that the lesser number of wind receptors in gregarious locusts is compensated by a higher frequency of spikes in the TCG to the same wind stimulus. However, because there is no difference in the signalling of the TCG in the two phases preceding flight initiation, an explanation of the lower behavioural threshold in gregarious locusts must lie in the actions of other interneurons.

How changes in the properties and actions of neurons from one phase to another are manifested in distinctly different movements is well illustrated by a shift in the properties of a resistance reflex at a leg joint that appears to underlie some of the differences in walking gait [28] (Figure 2E). During a step by a solitarious locust, the excursion of the hind femorotibial joint is smaller and the joint is kept more flexed compared with a gregarious locust (Figure 2F). This joint is extended during walking by the action of one excitatory motor neuron, the slow extensor tibiae (SETi). During static posture, the same motor neuron maintains the joint position under changing loads and in the face of external perturbations. The SETi spikes tonically in solitarious locusts, and its frequency increases at more extended angles, whereas in gregarious locusts it shows little tonic spiking regardless of the joint angle. In both phases, imposed flexion of the joint elicits resistance reflexes in the SETi, but regardless of the initial and final position of the leg, its spiking rate in solitarious locusts is at least twice that in gregarious locusts (Figure 2G). This increased sensory-motor gain in the neuronal networks controlling postural reflexes in solitarious locusts allows finer control of the rate and magnitude of movement and appears to be linked to the slow gait as well as to a behavioural catalepsy [28] in this phase.

### Evoking phase transitions

Despite the sheer scale of the long-term differences in brain structure and function between phases, key changes from solitarious to gregarious behaviour take just a few hours, particularly with regard to the critical decision whether to join or avoid other locusts. Other changes, such as in colour occur over a lifetime, and some morphological changes take several generations to be fully expressed [15,29]. All these slower changes pivot, however, on the decision to join the group and the ensuing rapid behavioural transition to gregarious-like behaviour. Everything else then follows upon positive sensory feedback from other locusts. A major focus of phase change research has therefore been to gain understanding of the molecular and cellular mechanisms underlying this initial behavioural change.

**Figure 3:**
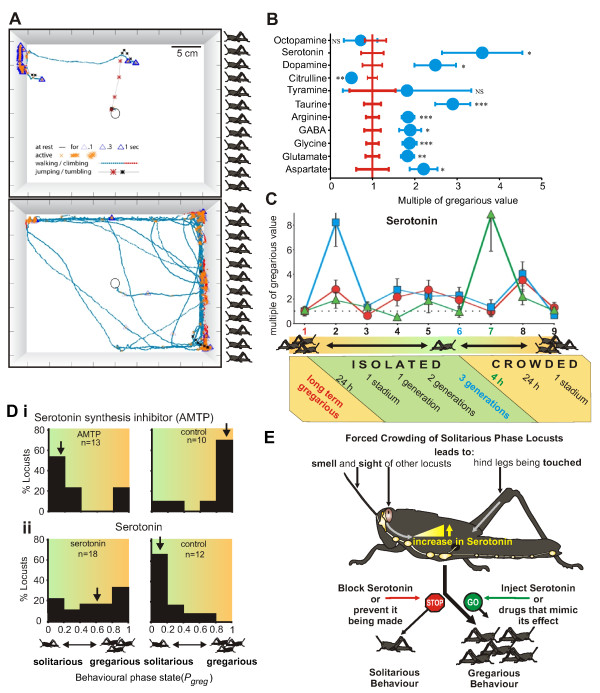
**Changes from solitarious to gregarious behaviour occur rapidly and are mediated by serotonin**. **(A) **Behaviour of a solitarious locust (top) and a gregarious locust (bottom) in the test arena is shown. A group of gregarious locusts is presented behind a clear, perforated wall to the right. Shown are representative tracks over ten minutes. After an initial jump, the solitarious locust moves slowly away from the group of locusts and spends a large proportion of the time motionless. The gregarious locust covers much more ground, spends a significant period close to the locust group and is rarely still. **(B) **Long-term solitarious and long-term gregarious locusts differ markedly in the amounts of key neurotransmitters and neuromodulators found in the CNS. Data from solitarious locusts (blue) are expressed as multiples of the amount found in gregarious (red) CNS (error bars are standard errors of the mean). **(C) **Time course of changes in serotonin in different brain regions during the entire phase change process, from the initial separation of long-term gregarious locusts through up to three generations of isolation, followed by increasing durations of crowding of long-term solitarious locusts; optic lobes (blue), central brain (red), thoracic ganglia (green). Data for **(B) **and **(C) **are from [36]. **(D) **Serotonin is (i) necessary and (ii) sufficient to induce behavioural gregarization. Histograms showing proportions of locusts displaying fully solitarious (*P*_greg _= 0 to 0.2) through to fully gregarious (*P*_greg _= 0.8 to 1) behaviour as measured in the arena (A). In (i), locusts were injected with the serotonin synthesis inhibitor α-methyl tryptophan (AMTP) or a saline control and then subjected to gregarizing stimuli for two hours; AMTP-treated locusts remained solitarious, unlike the controls. In (ii), serotonin or saline was topically applied to the thoracic ganglia for two hours in the complete absence of gregarizing stimuli; serotonin promoted gregarious behaviour. **(E) **Diagrammatic summary of the behavioural gregarization pathway and the role of serotonin as revealed by pharmacological manipulations such as those shown in **(D)**.

A necessary first requisite, however, has been the development of analytical techniques to quantify the differences in solitarious and gregarious behaviour. Detailed observations of individual locusts are made in a rectangular arena: at one end is a group of gregarious locusts behind a clear, perforated partition, and at the other is an equivalent but empty chamber (Figure 3A). Based on such analyses of the behaviour of 200 locusts of known phase (reared in crowded or isolated conditions for many generations), a binary logistic regression model is built which coalesces disparate behavioural characters into a single metric (*P*_greg_) that defines the behavioural phase state [15,16,30]. A *P*_greg _of 0 means that a locust behaves solitariously, whereas a *P*_greg _of 1 indicates fully gregarious behaviour. This statistical model can then be applied to locusts of unknown behavioural phase state to determine their *P*_greg _on the basis of their behaviour when introduced into the arena. In this way, transitional states between the two extremes can be quantified so that a direct link can be made between changes in the central nervous system (CNS) and behaviour.

If solitarious locusts are forced into a crowd so that they cannot escape from each other, then there is a rapid reconfiguration of behaviour towards gregariousness [31]. Locusts crowded for just two to four hours behave indistinguishably in the arena from locusts that have been gregarious for many generations. There are two distinct but equally effective sensory pathways that drive this rapid change [16,30]. First, the combined sight and smell of other locusts (and both kinds of stimulus must be present, or no behavioural change occurs) [32,33]: the cerebral pathway. Second, repeated stimulation of specific hind leg mechanoreceptors, which can be induced experimentally with a small paintbrush or naturally by jostling with other locusts [34,35]: the thoracic pathway. The connections that the sensory neurons of these receptors make with particular elements of the local circuits that control limb movements are known in detail [18].

### Chemical changes in the brain

An invaluable insight into the effect crowding or gregarizing stimuli have on the CNS was provided by a study analysing changes in the neurochemistry of the CNS as locusts change from one phase to the other [36]. The amounts of 13 potential neurotransmitters and/or neuromodulators were measured by performing high-performance liquid chromatography in different parts of the CNS of both long-term solitarious and long-term gregarious locusts, as well as in locusts undergoing phase transition. Eight of these substances were more abundant in both the brain and the thoracic ganglia of long-term solitarious locusts than in long-term gregarious ones, and three were less abundant (Figure 3B).

Crowding larval solitarious locusts led to rapid changes in six of these substances within the first 24 hours, by which time gregarious behaviour is already being expressed (Figure 3C). Only serotonin, however, showed a dramatic increase in the first four hours of crowding, the critical period during which gregarious behaviour is established. This increase was confined to the thoracic ganglia and could be induced by either the thoracic or the cerebral gregarizing pathway [30].

After crowding solitarious nymphs for a whole larval stage, the amounts of all chemicals except octopamine were similar to those of long-term gregarious locusts. The converse process of isolating larval gregarious locusts also led to rapid changes in seven chemicals equal to or even exceeding the differences seen between long-term solitarious and gregarious locusts. These measurements show that the levels of many neuroactive substances in the CNS change and reflect the time course of behavioural and physiological phase change. The rapid transient increase in serotonin precedes these changes during behavioural gregarization and led us to a closer examination of its role.

### Serotonin and the transition to gregarious behaviour

A series of experiments have shown that serotonin is both necessary and sufficient to induce initial behavioural gregarization [30] (Figures 3D and 3E). First, the extent of gregarious behaviour induced by different periods of crowding was positively correlated with the amount of serotonin in the thoracic ganglia. Locusts showing the most gregarious behaviour had three times the amount of serotonin than those showing solitarious behaviour. Second, injecting the serotonin receptor antagonists ketanserin and methiothepin into the thoracic CNS prevented the acquisition of gregarious behaviour. Inhibition of serotonin synthesis with α-methyltryptophan, a competitive antagonist of tryptophan hydroxylase, had the same effect (Figure 3D(i)). Third, topical application of serotonin onto the thoracic ganglia induced gregarious behaviour (Figure 3D(ii)). The serotonin receptor agonists α-methylserotonin and 5-carboxamidotryptamine also caused a significant shift towards gregarious behaviour. In both these experiments, the locusts had never encountered another locust prior to be being placed in the test arena. Finally, facilitating endogenous serotonin synthesis by the injection of the serotonin precursor 5-hydroxytryptophan amplified the effect of gregarizing stimuli presented for a brief period.

Once gregarious behaviour is acquired, however, it is not maintained by serotonin. The initial spike of serotonin decays within 24 hours, so long-term gregarious locusts have less than half the amount of serotonin in their CNS compared with long-term solitarious locusts. Moreover, gregarious behaviour maintains the gregarious phenotype but is itself highly labile. Newly gregarious locusts that have been crowded for 24 hours lose their gregarious behaviour after only 4 hours of reisolation [31], but locusts that have been in the gregarious phase for many generations only become behaviourally solitarious after days of isolation. This suggests that the long-lasting reinterpretation of the genome that is evident in the somatic phenotype is controlled and maintained through this highly labile behavioural phenotype. Whilst the arena is invaluable for evaluating overall activity and the attraction or repulsion of locusts to or from each other, it is not intended to encapsulate all the behavioural differences between phases. Some of these may have a rapid onset that cannot be detected in the assay, whilst others may change more slowly and constitute further tranches of phenotypic change. Serotonin clearly has an important role in initiating the conditions for behavioural change, but this does not exclude the possibility that other mechanisms might operate in parallel to effect other phase characters.

Analysis of the patterns of gene expression over the time courses of solitarization and gregarization in another species, the migratory locust (*Locusta migratoria*), have revealed rapid expressional changes in 1,444 (15.8%) of 9,154 genes analyzed. Genes implicated in peripheral odourant reception feature strongly among them, including genes that encode chemosensory proteins (CSP) and takeout (TO) proteins. Of these, the CSP gene *LmigCSP3 *and the TO gene *LmigTO1 *are implicated in the behavioural change between avoidance of and attraction to conspecifics by regulating peripheral odour sensitivity [37]. As in *S. gregaria*, these two transitions have different time courses, but in this species behavioural gregarization is slower than solitarization and was never fully achieved in these experiments. A second and contemporaneous report by the same group [38] identified *pale*, *henna *and *vat1*, genes involved in dopamine biosynthesis, synaptic release and cuticular melanisation, as critical targets related to behavioural phase changes. Moreover, injection of dopamine or a dopamine agonist is said to initiate gregarious behaviour as well as serotonin agonists. It remains uncertain how these separate central and peripheral mechanisms are interrelated to bring about the behavioural changes described in *Locusta*.

## Conclusions

How does serotonin exert its effect on behavioural gregarization in *S. gregaria*? Serotonin is a well-studied molecule, not least for its role in causing neuronal plasticity. Our studies suggest that serotonin may exert its effect via an adenylyl cyclase/protein kinase A pathway that is implicated in many classical learning paradigms. In locusts, it appears that a mechanism for inducing focal neuronal plasticity has become co-opted into a much more extensive reorganization of behaviour. More generally, however, serotonin is implicated in many forms of behavioural plasticity associated with social interactions with members of the same species. This ranges from the setting up of dominance hierarchies and the regulation of aggressive behaviour in crickets, crayfish and rodents through to emotional well-being and clinical depression in humans. The common thread is that serotonin plays a key role in modifying suites of behaviour in response to specific social signals. Phenotypic plasticity is driven by experience but may in turn change future environmental input. Altered behaviour in particular can remove the individual from or further expose the individual to the stimuli that caused the change, as well as to a host of other, associated stimuli. Moreover, changes may encompass the very sensory systems by which the animal senses its environment and hence how it detects and interprets that experience. The presence and/or salience of different environmental cues may therefore change dramatically. If these cues are important in the induction of the epigenetic change in the first instance, then feedback loops, either positive or negative, that drive further phenotypic change or normalise the phenotype may be set up. These environmental feedback systems mirror and extend the developmental switch and canalization mechanisms, respectively, that operate within gene regulatory networks. In clinical depression, or its 'learned helplessness' analogues in animal models, positive feedback loops driven by altered perception may reinforce behaviour that eventually becomes counterproductive.

The analysis of phase change in locusts provides us with insight into the reciprocal interactions between the environment and mechanisms of phenotypic plasticity that can build up to devastating locust swarms. More generally, it gives us deep insights into the neurobiology of social interaction.

## Competing interests

The authors declare that they have no competing interests.

## Authors' contributions

All authors contributed equally to the writing of this review and read and approved the final manuscript.
